# Prediction of Hope and Morale During COVID-19

**DOI:** 10.3389/fpsyg.2021.739645

**Published:** 2021-09-22

**Authors:** Shaul Kimhi, Yohanan Eshel, Hadas Marciano, Bruria Adini

**Affiliations:** ^1^Stress and Resilience Research Center, Tel-Hai College, Tel Hai, Israel; ^2^Department of Psychology, University of Haifa, Haifa, Israel; ^3^The Ergonomics and Human Factors Unit, University of Haifa, Haifa, Israel; ^4^Department of Emergency and Disaster Management, School of Public Health, Sackler Faculty of Medicine, Tel Aviv University, Tel Aviv, Israel

**Keywords:** hope, morale, COVID-19, individual resilience, community resilience, national resilience

## Abstract

The current study uses a repeated measures design to compare two-time points across the COVID-19 pandemic. The first was conducted at the end of the “first wave” [T1] and the second was carried out on October 12-14 2020 (the last period of the second total general lockdown) in Israel. The participants (*N* = 805) completed the same questionnaire at both time points. The study examined the predictions of hope and morale at T2 by psychological and demographic predictors at T1. Results indicated the following: (a) The three types of resilience (individual, community, and national) significantly and positively predicted hope and morale. (b) Well-being significantly and positively predicted hope and morale. (c) Younger age significantly and positively predicts higher hope, but not morale. (d) A higher level of religiosity significantly and positively predicts higher hope and morale. (e) More right-wing political attitudes significantly and positively predict higher hope, but not moral. (f) More economic difficulties due to the pandemic, significantly and negatively predict hope and morale. We concluded that hope and morale can serve as significant indicators of the population's ability to cope with the COVID-19 pandemic. Furthermore, they can serve as a “thermometer” for the general mood of the population and can be used by decision-makers to assess coping ability at varied stages of the pandemic.

*We have always held to the hope, the belief, the conviction that there is a better life, a better world, beyond the horizon*.

Franklin D. Roosevelt

## Introduction

The COVID-19 pandemic originated in China toward the end of 2019 and in a short time spread to 213 countries and territories [World Health Organization (2019)]. The degree of pandemic damage varies from country to country, but it can be stated that it is an unprecedented threat in its scope and degree of damage affecting many areas of normal life (Anderson et al., 2020; Wang et al., 2020). This pandemic has severely disrupted the proper functioning of the global community, leading to the closure of schools and academic institutions, partial or complete lockdowns, reduced public transportation and aviation, unemployment and economic hardships, the decline of global stock markets, and panic shopping due to widespread concerns about supply shortages (Anzai et al., 2020). The purpose of the current study is to examine to what degree psychological and demographic characteristics measured at a relatively successful stage of the COVID-19 (end of “the first wave,” T1) predict the level of hope and morale toward the end of the “second lockdown” (T2) among the Israeli population. To the best of our knowledge, no study examined the effect of COVID-19 on the level of morale and hope using a longitudinal design.

### Resilience

A literature review indicates a considerable number of definitions for the concept of resilience. For example, the American Psychological Association defines resilience as a process of bouncing back from difficult experiences and adapting well in the face of adversity, trauma, tragedy, threats, or significant sources of stress (APA.org, 2014). Masten (2018) defines resilience as “the potential of the manifested capacity of a dynamic system to adapt successfully to disturbances that threaten the function, survival, or development of the system,” (p. 187). Beyond the various definitions, it seems that researchers agree on several areas: the concept of resilience has often been used in discussing people's ability to withstand stress and adversity (Bonanno, 2004; Ajdukovic et al., 2015); resilience is a complex multifaceted concept whose measurement arouses a rich debate (e.g., Bonanno et al., 2015); resilience is defined differently in the context of individuals, families, organizations, societies, and cultures (Southwick et al., 2014).

Three types of resilience have been extensively studied: individual, community, and national resilience. (a) According to Cacioppo et al. (2011), *individual resilience* is “the capacity to foster, engage in, and sustain positive relationships and to endure and recover from life stressors and social isolation” (p. 44). Bonanno et al. (2015) report that individual resilience contributes significantly and negatively to the prediction of depression, anxiety, stress, and obsessive-compulsive symptoms. According to Chen and Bonanno (2020), despite the serious nature of the COVID-19 pandemic, most individuals are likely to be resilient, as is true for other adversities. (b) Based on the above researchers, *community resilience* expresses the interaction between individuals and their community and refers to the success of the community in providing for the needs of its members and the extent to which individuals are helped by their community. (c) Other researchers define *national resilience* as a broad concept addressing issues of social sustainability and strength in several diverse realms: trust in the integrity of the government, the parliament, and other national institutions, belief in social solidarity, and patriotism (Ben-Dor et al., 2002).

### Subjective Wellbeing

Subjective wellbeing was defined by Diener (2006) as “An umbrella term for different valuations that people make regarding their lives, the events happening to them, their bodies and minds, and the circumstances in which they live” (P. 400). Later on Diener et al. (1999) claimed that well-being can be broken down into emotional, social, and psychological well-being. The concept of well-being is defined in the research literature in various forms and usually refers to sensors like happiness, and positive affect (Naci and Ioannidis, 2015). Fredrickson (2001) explained the positive association between well-being and hope: positive emotions contribute to broader subjective well-being because people who feel good are usually more open to new situations, relationships and impressions and therefore gain more experience and skills.

Studies that examined the effect of COVID-19 indicated that it impaired the standard of living due to various limitations, resulting from attempts to combat the pandemic (Qiu et al., 2020). The current study examines subjective wellbeing at T1 as a predictor of hope and morale at T2, during the COVID-19 pandemic.

### Hope

There are many different definitions to the concept of hope (Bruininks and Malle, 2005; Luo et al., 2020). According to Webster Dictionary, to hope means to cherish a desire with anticipation: to want something to happen or be true (https://www.merriam-webster.com/dictionary/hope?src=search-dict-box). Hope is probably best conceptualized by Snyder et al. (1991) as “a positive motivational state that is based on an interactively derived sense of successful (a) agency (goal-directed energy) and (b) pathways (planning to meet goals).” While Snyder emphasizes the cognitive aspect of hope, other researchers (e.g., Fredrickson, 2001) refer to its emotional aspects. Research claimed that parental attachment, stressful life events, and personality variables are likely to explain the origins of hope (Otis et al., 2016).

Beyond the various definitions and methods of measurement, there is agreement of different researchers concerning the positive effect that hope may have in many situations (e.g., Cavanaugh et al., 2015) and disasters (Thornton, 2020).

The association between hope and individual resilience has been explored in several studies. Previous research has shown a positive relationship between these two variables (Morote et al., 2017). Luthans et al. (2010) claimed that hopeful individuals possess positive thinking as well as the belief that they can produce routes to desired goals. Such individuals take a resilience stance by perceiving obstacles as challenges to overcome and by utilizing their optimism to plan alternatives to achieve their end goal. Schneider (2001) as well as Arampatzi et al. (2019) have argued further that hope and resilience are closely aligned constructs, as they both include a tendency toward maintaining an optimistic outlook in the face of adversity. To the best of our knowledge, no previous study examined the impacts of individual, community, and national resilience on hope and morale in times of a pandemic. However, based on the available research, we assume that these three resiliencies will positively predict both hope and morale.

### Morale

Webster Dictionary defines morale as “the mental and emotional condition (as enthusiasm, confidence, or loyalty) of an individual or group concerning the function or tasks at hand.” Although morale is somewhat an elusive concept, it was also defined as “a quality which involves feelings, emotions, attitude and perception toward the organization and its members. Positive morale is usually characterized by discipline, confidence and willingness to perform” (Shaban et al., 2017). The concept of morale emerged in the military setup (also known as “esprit de corps,” US Army, 1983). It is defined in terms of the mental, emotional, and spiritual state of the individual rather than in terms of a personality attribute. According to Din and Khuwaja (2016) high morale refers to the adjectives of happy, confident, appreciated, whereas sad, depressed, and unrecognized are related with low morale. The role of morale as a distress-reducing factor was investigated in the military context. An analysis of several modern wars (Gal and Mangelsdorff, 1991) concluded that when a military force fostered high morale among its troops, it was less likely to suffer a substantial number of distress casualties. Therefore, we suggest that morale can be viewed as a future-oriented perspective regarding the challenges of coping with one's current situation. A higher level of morale is likely to be associated with a more positive future orientation and with better resilience in hard times. Due to the strong association between morale and hope (Kimhi et al., 2020a) we assume that morale will be predicted as well by the three measures of resilience.

Morale research seems to focus on a particular group that is at the center of the struggle with a crisis, such as the military during the war (Johannesson, 2020), or a hospital during a health crisis (Garrett and McNolty, 2020). In the present study, we referred to morale as a general measure of mood during the COVID-19 pandemic among a large sample of the Israeli population. Specifically, we assumed that hope represents future expectations, while morale represents the present condition.

The present study examines resilience, wellbeing, and demographic characteristics as potential predictors of hope and morale during the COVID-19 pandemic. In a previous study, based on the two measurements (Kimhi et al., 2020b) we reported, among others, a significant decline of hope and morale, comparing a measurement taken at the end of the “first wave” (May 4–7, with the release from a full lockdown that was imposed on the Israeli residents; henceforth T1) and the second measurement took place at the beginning of a “second wave,” (July 12–15, when the rising numbers of confirmed COVID-19 patients increased the probability of the re-issuing of restrictive measures to combat the pandemic, including renewed lockdowns; henceforth T2). In the current study, we assumed that all three resilience types (individual, community, and national), as well as well-being measured at T1, will positively predict hope and morale measured at T2.

### Demographic Characteristics

A study of coping with old age (Moraitou et al., 2006) has found that age, marital status, and place of residence were moderately related to hope, and that this finding is in agreement with previous research (Cheavens and Gum, 2000). A more recent study (Kimhi et al., 2020d) have found that age and family income negatively predicted distress symptoms and sense of danger, whereas being a woman and economic difficulties positively predicted these two variables. There is reason to believe that these predictors will reversely predict hope and morale, which were negatively correlated with distress and sense of danger in this study.

Earlier studies have indicated that higher levels of religiosity and more right-wing political attitudes in Israel, significantly and positively predicted resilience while economic difficulties due to the COVID-19 pandemic, significantly and negatively predicted resilience (Kimhi et al., 2020c). The Israeli society consists of five main levels of religiosity, from secular to very religious (ultraorthodox) (Keshet and Popper-Giveon, 2021). A previous study identified a higher level of coherence and additional components of resilience amongst ultraorthodox respondents compared to secular respondents (Braun-Lewensohn et al., 2020). Based on these results, regarding demographic characteristics we assumed that these demographic characteristics would also predict hope and morale at T2.

Based on the existing literature regarding hope and morale during the COVID-19 pandemic, we hypothesized that psychological and demographic characteristics, controlling each other, in T1 will significantly predict hope and morale, controlling each other, in T2 as following: (a) Individual, community, and national resilience, and subjective wellbeing will significantly and positively predict hope and morale: the higher resilience and wellbeing, the higher hope and moral reported. (b) Age, level of religiosity, and political attitudes will significantly and positively predict hope and morale The older the age, the higher level of religiosity, and the more right-wing political attitudes, the higher hope and morale report. On the other end, economic difficulty due to the pandemic will significantly and negatively predict hope and morale.

## Method

### Study Design

This study is a longitudinal study, based on two repeated measurements (the same sample of respondents answered the same questionnaire in two different measurements), performed on a large sample of respondents in Israel. The first measurement (T1) was carried out in early May 2020 (May 4–7), when the first wave of the pandemic seemed to recede, and the full lockdown and other restrictions on the population were lifted. The second measurement (T2) was conducted in midst of the second lockdown (October 12–14). In this study we examined four psychological (individual, community, and national resilience, and subjective wellbeing), and four demographic (age, level of religiosity, political attitudes, and economic difficulty due to the pandemic) characteristics at T1 predicting hope and morale at T2.

### Participants

The data was collected by an internet panel company that consists of over 65,000 panelists, representing all demographic sectors and geographic locations in Israel (https://sekernet.co.il/). The sample included 906 Jewish Israeli respondents, who answered two times an online questionnaire. The questionnaire was approved by the Ethics Committee of Tel Aviv University, and an informed consent form was signed by all participants. The demographic and psychological characteristics of the sample are detailed in Table 1.

**Table 1:** Demographic characteristics of the respondents (*N* = 906).

**Variable (T1)**	**Group**	**No. of respondent**	**%**	**Mean (S.D)**
Age	18–30	211	23	44.08 (15.53)
	31–40	212	23	
	41–60	164	18	
	51–60	156	17	
	61–70	125	14	
	71 on	38	4	
Level of religiosity	Secular	437	48	
	Traditional	269	30	
	Religious	117	13	
	Very religious (ultra orthodox)	83	9	
Political attitudes	1. Extreme left	10	1	3.50 (0.85)
	2. Left	99	11	
	3. Center	323	34	
	4. Right	381	44	
	5. Extreme right	93	9	
Economic difficulties due to the pandemic	1. Not at all	176	19	2.72 (1.24)
	2. A little	239	26	
	3. Medium	247	27	
	4. Much	150	17	
	5. Very much	94	10	

### Study Tools

#### Level of Hope

This tool, which was constructed specifically for the present study, is based on an earlier scale (Jarymowicz and Bar-Tal, 2006; Halperin et al., 2008), that was designed to measure the level of hope for peace between Israel, the Arab nations, and the Palestinians. Its two dimensions are personal and collective hope. The current scale of hope, in the context of coronavirus/COVID-19 pandemic, includes five items. Two of them refer to the personal level (e.g., “I hope that I will emerge strengthened from the coronavirus/COVID-19 crisis”) and three items refer to the collective level (e.g., “I hope that Israeli society will emerge strengthened from the coronavirus/COVID-19 crisis”). This measurement tool was found in a previous study to be valid (Kimhi et al., 2020a). The response scale ranged from 1 = very little hope to 5 = high hope. The internal reliability of the scale in the present study was found to be high across the three measurements (α = 0.92).

#### Morale

The level of personal morale was examined by a single item: “How would you define your morale these days?” The response scale ranges from 1 = not good at all, to 5 = very good. It should be noted that young Israeli adults serve in the IDF and continue serving in the reserve forces. Later on, they share their children's experiences as soldiers. The concept of morale accompanies, therefore, most of the members of the present sample. This item was found in a previous study to be valid (Kimhi et al., 2020a).

#### National Resilience

The original national scale (Kimhi and Eshel, 2019) includes 13 items, whereas the scale in the present study includes 16 items. The three additional items pertain specifically to the COVID-19 crisis. Examples of the original scale items include: “In a national crisis, the Israeli society will stand behind the decisions of the government and its leader” and “Israel is my home and I do not intend to leave it.” An example of a new item is: “I have full confidence in the ability of the Israeli healthcare system to take care of the population during the coronavirus/COVID-19 crisis.” The response scale for the national resilience items ranges from 1 = do not agree at all to 6 = strongly agree. The internal reliability of the scale was high across the two measurements (α = 0.91).

#### Community Resilience

This resilience scale includes 10 items that relate to the subjects' identification with their community and their confidence in their ability to cope with the difficulties they will face (Leykin et al., 2013). Responses to the questionnaire items represent a 5-point scale, ranging from 1 = do not agree at all, to 5 = agree to a very large extent. Examples of items: “The municipal authority in my locality is functioning properly in the coronavirus/COVID-19 crisis,” “I can trust people in my locality to come to my aid in case of a crisis, including the coronavirus/COVID-19 crisis.” The current study's internal scale reliabilities were high across the two measurements (α = 0.93 at T1 and α = 0.94 at T2).

#### Individual Resilience

The short version of this questionnaire (Campbell-Sills and Stein, 2007) includes 10 items about a sense of personal resilience in face of difficulties (Connor and Davidson, 2003). Examples of questions: “I am able to adapt when changes occur”; “I am not easily discouraged by failures.” Responses to the questionnaire items are ranked using a 5-point scale ranging from 0 = not true at all, to 4 = true nearly all the time. In the present study, the internal reliability of the scale was high across the two measurements: α = 0.89 (T1, and T2).

#### Subjective Well-Being

This scale consists of nine items concerning individuals' perception of their lives in the present regarding various contexts, such as work, family life, health, free time, and others. This scale was validated and successfully employed in previous studies (e.g., Kimhi and Eshel, 2009; Eshel et al., 2016; Kimhi et al., 2017). Responses to these items range from 1 = very bad to 6 = very good. Example: “How is your health today?” Its reliabilities in the present study were found to be good in both measurements (α = 0.87 at T1 and α = 0.86 at T2).

### Demographic Characteristics

Four demographic characteristics were examined in the current study: Age (18–30, 31–40, 41–60, 60+). Religiosity was determined by a four-point item: “Please indicate whether you regard yourself as 1. Secular, 2. Traditional, 3. Religious, 4. Very religious (ultraorthodox).” The political attitude was determined by a five-point item: “Please indicate whether your political attitudes are 1. Extreme left, 2. Left, 3. Center, 4. Right, 5. Extreme right.” Economic difficulties due to the pandemic to the respondent or his/her family were rated by a five-point scale: 1 = not at all, 5 = very much.

**Figure 1:**
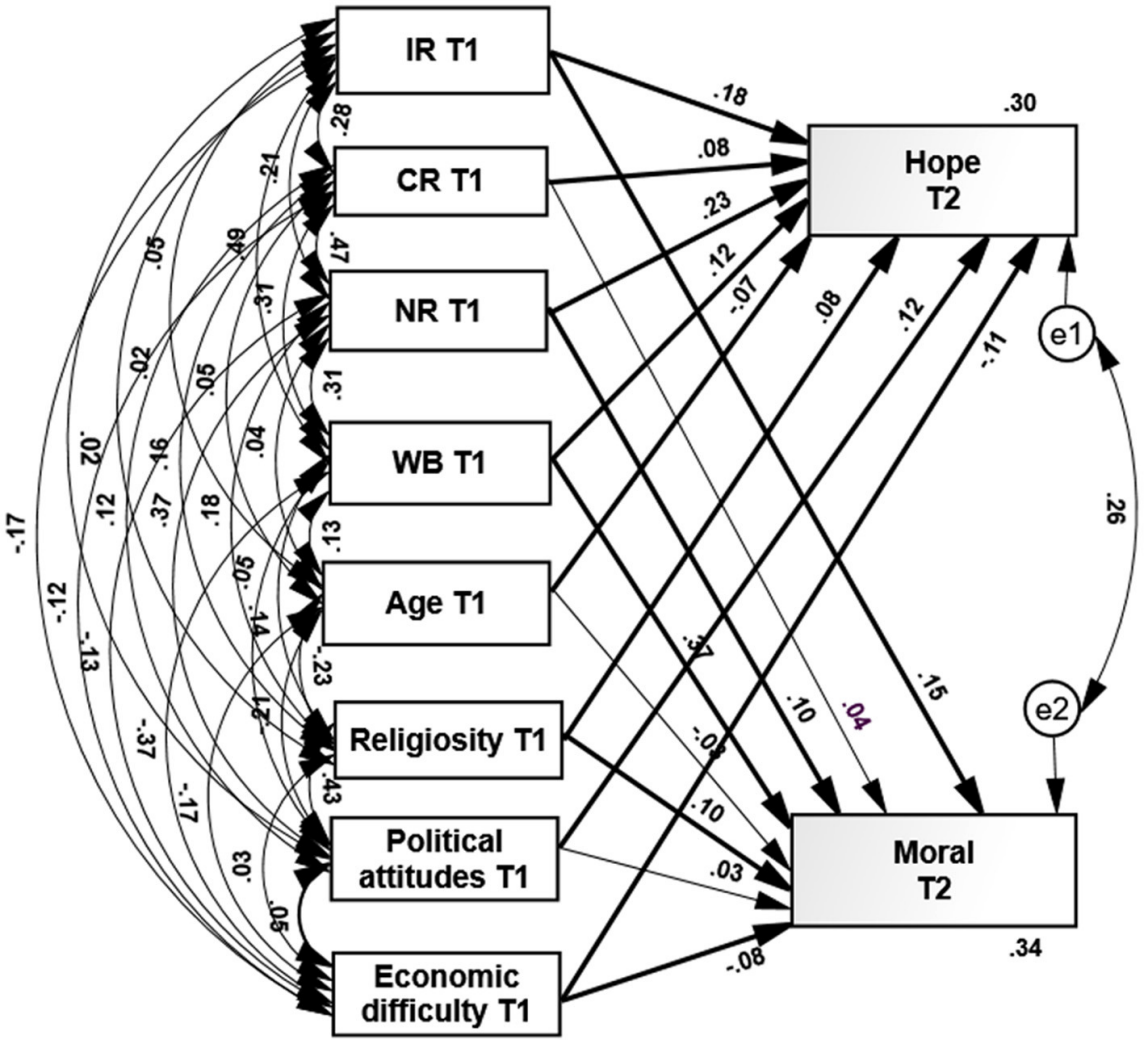
Path analysis of psychological and demographic variables at T1 predicting hope and morale at T2. IR, individual resilience; CR, Community resilience; NR, National resilience; WB, Wellbeing. The thick lines indicates significant paths, the thin lines indicate non-significant paths.

To examine our two hypotheses regarding the prediction of hope and morale at T2 by three types of resilience, subjective well-being, and demographic characteristics, we calculated path analyses (Arbuckle, 2009) using the Amos software (Table 2; Figure 1).

**Table 2:** Path analysis of psychological and demographic variables at T1 predicting hope and morale at T2.

**Predictors**	**Predicted**	
	**Hope T2**	**Morale T2**
Individual resilience	0.179^***^	0.155^***^
Community resilience	0.085^**^	0.041^**^
National resilience.	0.227^***^	0.097^**^
Subjective wellbeing	0.118^***^	0.375^***^
Age	−0.066^*^	0.028
Level of religiosity	0.080^*^	0.097^**^
Political attitudes	0.122^***^	0.026
Economic difficulty due to pandemic	−0.107^***^	−0.076^**^
Explained variance (*R*^2^)	0.30	0.34

* *p < 0.05*,

** *p < 0.01*,

*** *p < 0.001*.

## Results

Results regarding psychological characteristics (Table 2; Figure 1), indicate the following: (a) Individual, community, and national resilience at T1 significantly and positively predicted hope and morale in T2: The higher the resilience, the higher hope and morale reported. Furthermore, national resilience at T1 was the best predictor of hope at T2. These results mainly support our first hypothesis. (b) Subjective well-being (SWB) at T1 significantly and positively predicted hope and morale at T2: The higher SWB at T1, the higher hope and morale reported at T2. Additionally, SWB at T1 was the best predictor of morale at T2. These results fully support our first hypothesis regarding SWB. Overall, psychological predictors at T1 explained 25% of hope and 28% of morale variance. These results mainly support our first hypothesis.

Results regarding demographic characteristics indicated the following: (a) According to our hypothesis, age-predicted a significantly level of morale: The older age, the higher morale reported. However, in contrast with our second hypothesis, age did not significantly predict hope. (b) In line with our second hypothesis, the level of religiosity significantly and positively predicted hope and morale: The higher level of religiosity, the higher levels of hope and morale reported at T2. (c) In line with our second hypothesis, political attitudes significantly predicted hope and morale: The more right-wing political attitudes, the higher hope, and morale reported at T2. (d) In line with our second hypothesis, economic difficulties due to the pandemic at T2 significantly and negatively predicted hope and morale at T2: The higher the economic difficulties, the lower level of hope and morale reported at T2. The eight predictors explained 30% of hope and 34% of morale variance in T2. These results mainly supported our second hypothesis.

## Discussion

The current research is based on two repeated measurements during the COVID-19 pandemic and examined psychological and demographic characteristics measured at T1 predicting hope and morale measured at T2. The psychological characteristics included four predictors: individual, community, and national resilience, and subjective wellbeing, as predictors of hope and morale. The demographic characteristics included four demographic characteristics (age, level of religiosity, political attitudes, and economic difficulty due to the pandemic), as predictors of hope and morale.

The results of the indicated that the four psychological variables significantly and positively predicted the level of hope and morale. It can also be argued that the psychological variables examined, better explain hope and morale, compared to the demographic variables examined. These results support previous studies that indicated that a higher level of hope and morale characterize people who can better cope with adversities, compared to people with a lower level of hope and morale (Cavanaugh et al., 2015; Johannesson, 2020; Thornton, 2020). Additionally, these results are consistent with the findings of earlier studies showing that higher resilience better predicts coping with distress (e.g., Bonanno et al., 2015; Kimhi et al., 2020c). It is possible to claim, based on our study, that the level of resilience at the first stage of adversity, may be associated with a higher level of hope and morale at a later stage of the adversity. However, since the contribution of the three types of resilience to hope and morale has hardly been explored before, further studies are needed to support the currently found associations.

The best predictor of hope is national resilience. This result support earlier study result (Kimhi et al., 2020c). However, it is worth noting that community resilience was the lowest predictor of hope and morale, among the three types of resilience. This finding differs from earlier findings that dealt with resilience in the context of security and terrorism threats, in which we found that community resilience has a very important role in the individual's coping with the crisis (e.g., Kimhi et al., 2020c). This finding may be related to the fact that in the current health crisis, other people of the community may pose a life-threatening risk rather than a source of solidarity and consolation, which probably have characterized other crises, such as war or terrorism. It is also possible that the restrictions imposed on the public in terms of 'social distance', closure, etc. reduce the importance of the community concerning morality and hope, and hence they have a lesser effect in a pandemic outline on the individual's ability to cope in a crisis.

The result of our study, regarding the prediction of hope and morale by demographic characteristics, indicated that the best predictor of hope at T2 was political attitudes at T1. These results support an earlier study indicating the central role of political attitudes in coping with this pandemic in Israel (e.g., Kimhi et al., 2020d). Level of religiosity and economic difficulties were the best predictors of morale. The results regarding religiosity and morale, support other studies which pointed to the connection between religiosity and satisfaction and morale in a crisis (e.g., Gal and Mangelsdorff, 1991; Din and Khuwaja, 2016).

Several studies show that economic hardships affect humans in many ways, such as mental state (Gruber et al., 2020), social equality (Alon et al., 2020), and more. These studies finding indicates the importance of the economic factor that accompanies the coronavirus/COVID-19 pandemic, compared to other demographic characteristics such as age, political attitudes, and level of religiosity. The results of our research add to this list, by presenting the prediction of both hope and morale by economic difficulties. This finding is of importance to all decision-makers regarding coping and recovery from the COVID-19 pandemic.

### Conclusions and Limitations

Two notable limitations to this study are: the sample is based on a web sample and not on a random sample, and the other is the fact that this is a correlative study that does not allow inference to be derived. Despite these limitations, the findings of the present study suggest that hope and morale can serve as significant indicators of the population's ability to cope with the COVID-19 pandemic.

Hope and morale can serve as a “thermometer” for the general mood of the population and can be used by decision-makers to assess coping ability at varied stages of the crisis. Furthermore, as hope and morale were found to be associated with all types of resilience and well-being, we recommend that governance authorities invest efforts in enhancing them by empowering risk communication. This should be assessed in future studies to identify the degree of impact of such measures in relation to resilience of the population. Additionally, our results point to the importance of relating to the economic crisis that accompanies the pandemic and the vital need for the government's economic assistance to those affected (e.g., Pomerleau, 2020).

## Data Availability Statement

The datasets presented in this article are not publicly available due to the commitment of the research team to the subjects to complete anonymity and a commitment that the data will not be given to any other body, except the researchers. Requests to access the datasets should be directed to shaulkim@gmail.com.

## Ethics Statement

The studies involving human participants were reviewed and approved by Tel Aviv University ethic committee. The patients/participants provided their written informed consent to participate in this study.

## Author Contributions

All authors listed have made a substantial, direct and intellectual contribution to the work, and approved it for publication.

## Funding

This work was supported by Ministry of Science and Technology of the State of Israel.

## Conflict of Interest

The authors declare that the research was conducted in the absence of any commercial or financial relationships that could be construed as a potential conflict of interest.

## Publisher's Note

All claims expressed in this article are solely those of the authors and do not necessarily represent those of their affiliated organizations, or those of the publisher, the editors and the reviewers. Any product that may be evaluated in this article, or claim that may be made by its manufacturer, is not guaranteed or endorsed by the publisher.

## References

[B1] Ajdukovic D. Kimhi S. Lahad M. (eds.). (2015). Resiliency: Enhancing Coping With Crisis and Terroris. Amsterdam: IOS Press, The NATO Science for Peace and Security Programme.

[B2] Alon T. M. Doepke M. Olmstead-Rumsey J. Tertilt M. (2020). The Impact of COVID-19 on Gender Equality (No. w26947). Cambridge, MA: National Bureau of Economic Research.

[B3] Anderson R. M. Heesterbeek H. Klinkenberg D. Hollingsworth T. D. (2020). How will country-based mitigation measures influence the course of the COVID-19 epidemic? Lancet 395, 931–934. 10.1016/S0140-6736(20)30567-5 32164834 PMC7158572

[B4] Anzai A. Kobayashi T. Linton N. M. Kinoshita R. Hayashi K. Suzuki A.. (2020). Assessing the impact of reduced travel on exportation dynamics of novel coronavirus infection (COVID-19). J. Clin. Med. 9:601. 10.3390/jcm9020601 32102279 PMC7073579

[B5] APA.org (2014). The Road to Resilience. Available online at: http://www.apa.org/helpcenter/road-resilience.aspx (accessed September 2, 2020).

[B6] Arampatzi E. Burger M. Stavropoulos S. Tay L. (2019). The role of positive expectations for resilience to adverse events: subjective well-being before, during and after the Greek bailout referendum. J. Happiness Stud. 21, 965–995. 10.1007/s10902-019-00115-9

[B7] Arbuckle J. L. (2009). Amos 19. Crawfordville, FL: AMOS Development Corporation.

[B8] Ben-Dor G. Pedahzur A. Canetti-Nisim D. Zaidise E. (2002). The role of public opinion in Israel's national security. Am. Jewish Congr. Congr. Monthly 69, 13–15.

[B9] Bonanno G. A. (2004). Loss, trauma, and human resilience: have we underestimated the human capacity to thrive after extremely aversive events?” Am. Psychol. 59:20. 10.1037/0003-066X.59.1.20 14736317

[B10] Bonanno G. A. Romero S. S. Klein S. I. (2015). The temporal elements of psychological resilience: an integrative framework for the study of individuals, families, and communities. Psychol. Inq. 26, 139–169. 10.1080/1047840X.2015.992677

[B11] Braun-Lewensohn O. Abu-Kaf S. Kalagy T. (2020). Hope and resilience during a pandemic among three cultural groups in Israel: the second wave of Covid-19. Front. Psychol. 12:637349. 10.3389/fpsyg.2021.637349 33679564 PMC7930000

[B12] Bruininks P. Malle B. F. (2005). Distinguishing hope from optimism and related affective states. Motiv. Emot. 29, 324–352. 10.1007/s11031-006-9010-4

[B13] Cacioppo J. T. Reis H. T. Zautra A. J. (2011). Social resilience. Am. Psychol. 66, 43–51. 10.1037/a0021419 21219047

[B14] Campbell-Sills L. Stein M. B. (2007). Psychometric analysis and refinement of the Connor–Davidson resilience scale (CD-RISC): validation of a 10-item measure of resilience. J. Trauma. Stress 20, 1019–1028. 10.1002/jts.20271 18157881

[B15] Cavanaugh L. A. Bettman J. R. Luce M. F. (2015). Feeling love and doing more for distant others: specific positive emotions differentially affect prosocial consumption. J. Mark. Res. 52, 657–673. 10.1509/jmr.10.0219

[B16] Cheavens J. Gum A. (2000). “Gray power: hope for the ages,” in Handbook of Hope. Theory, Measures, and Applications, ed Snyder C. R. (San Diego, CA: Academic Press), 201–221.

[B17] Chen S. Bonanno G. A. (2020). Psychological adjustment during the global outbreak of COVID-19: a resilience perspective. Psychol. Trauma Theory Res. Pract. Policy 12:S51. 10.1037/tra0000685 32538658

[B18] Connor K. M. Davidson J. R. (2003). Development of a new resilience scale: the Connor-Davidson resilience scale (CD-RISC). Depress. Anxiety 18, 76–82. 10.1002/da.10113 12964174

[B19] Diener E. (2006). Guidelines for national indicators of subjective well-being and ill-being. J. Happiness Stud. 7, 397–404. 10.1007/s10902-006-9000-y

[B20] Diener E. Suh M. E. Lucas E. R. Smith L. H. (1999). Subjective well-being: three decades of success. Psychol. Bull. 25, 270–302. 10.1037/0033-2909.125.2.276

[B21] Din M. Khuwaja N. A. (2016). The interplay of emotional intelligence and morale of university teachers. Int. Res. J. Arts Hum. 44, 113–120. Available online at: https://sujo-old.usindh.edu.pk/index.php/IRJAH/article/view/2825

[B22] Eshel Y. Kimhi S. Lahad M. Leykin D. (2016). Individual, community, and national resiliencies and age: are older people less resilient than younger individuals? Am. J. Geriatr. Psychiatry 24, 644–647. 10.1016/j.jagp.2016.03.002 27160987

[B23] Fredrickson B. L. (2001). The role of positive emotions in positive psychology. The broaden-and-build theory of positive emotions. Am. Psychol. 56, 218–226. 10.1037/0003-066X.56.3.218 11315248 PMC3122271

[B24] Gal R. Mangelsdorff D. (eds.). (1991). “Cultural and societal factors in military organizations,” in The Handbook of Military Psychology (New York, NY: Wiley), 471–489.

[B25] Garrett J. R. McNolty L. A. (2020). More than warm fuzzy feelings: the imperative of institutional morale in hospital pandemic responses. Am. J. Bioethics 20, 92–94. 10.1080/15265161.2020.1779407 32716791

[B26] Gruber J. Prinstein M. J. Clark L. A. Rottenberg J. Abramowitz J. S. Albano A. M.. (2020). Mental health and clinical psychological science in the time of COVID-19: challenges, opportunities, and a call to action. Am. Psychol. 76, 409–426. 10.1037/amp0000707 32772538 PMC7873160

[B27] Halperin E. Bar-Tal D. Nets-Zehngut R. Drori E. (2008). Emotions in conflict: correlates of fear and hope in the Israeli-Jewish society. Peace Confl. J. Peace Psychol. 14, 233–258. 10.1080/10781910802229157

[B28] Jarymowicz M. Bar-Tal D. (2006). The dominance of fear over hope in the life of individuals and collectives. Eur. J. Soc. Psychol. 36, 367–392. 10.1002/ejsp.302

[B29] Johannesson J. (2020). The critical role of morale in ukraine's fight against the Russian invasion. Open J. Soc. Sci. 8:252. 10.4236/jss.2020.86022

[B30] Keshet Y. Popper-Giveon A. (2021). “I Took the Trouble to Make Inquiries, So I Refuse to Accept Your Instructions”: religious authority and vaccine hesitancy among ultra-orthodox jewish mothers in Israel. J. Relig. Health 60, 1992–2006. 10.1007/s10943-020-01122-4 33389435 PMC7778477

[B31] Kimhi S. Eshel Y. (2009). Individual and public resilience and coping with long-term outcomes of war. J. Appl. Biobehav. Res. 14, 70–89. 10.1111/j.1751-9861.2009.00041.x20494270

[B32] Kimhi S. Eshel Y. (2019). Measuring national resilience: a new short version of the scale (NR-13). J. Commun. Psychol. 47, 1–12. 10.1002/jcop.22135 30295954

[B33] Kimhi S. Eshel Y. Marciano H. Adini B. (2020a). A renewed outbreak of the COVID-19 pandemic: A longitudinal study of distress, resilience and subjective well-being. Int. J. Environ. Res. Public Health 17:7743. 10.3390/ijerph17217743 33113914 PMC7660159

[B34] Kimhi S. Eshel Y. Marciano H. Adini B. (2020b). Recovery from the COVID-19 pandemic: distress and resilience. Int. J. Disaster Risk Reduct. 50:101843. 10.1016/j.ijdrr.2020.101843 32953439 PMC7491376

[B35] Kimhi S. Eshel Y. Marciano H. Adini B. (2020c). Community and national resilience and their predictors in face of terror. Int. J. Disaster Risk Reduct. 50:101746. 10.1016/j.ijdrr.2020.10174632100155

[B36] Kimhi S. Marciano H. Eshel Y. Adini B. (2020d). Resilience and demographic characteristics predicting distress during the COVID-19 crisis. Soc. Sci. Med. 265:113389. 10.1016/j.socscimed.2020.113389 33039732 PMC7518838

[B37] Kimhi S. Eshel Y. Leykin D. Mooli Lahad M. (2017). Individual, community, and national resilience in peace time and in the face of terror: a longitudinal study. Pers. Individ. Dif. 114, 160–166. 10.1080/15325024.2017.1391943

[B38] Leykin D. Lahad M. Cohen O. Goldberg A. Aharonson-Daniel L. (2013). Conjoint community resiliency assessment measure-28/10 items (CCRAM28 and CCRAM10): a self-report tool for assessing community resilience. Am. J. Commun. Psychol. 52, 313–323. 10.1007/s10464-013-9596-0 24091563

[B39] Luo S. X. Van Horen F. Millet K. Zeelenberg M. (2020). What we talk about when we talk about hope: a prototype analysis. Emotion 1–18. 10.1037/emo0000821 32614195

[B40] Luthans F. Avey J. B. Avolio B. J. Peterson S. J. (2010). The development and resulting performance impact of positive psychological capital. Hum. Resour. Dev. Q. 21, 41–67. 10.1002/hrdq.20034

[B41] Masten A. S. (2018). Resilience theory and research on children and families: past, present, and promise. J. Fam. Theory Rev. 10, 12–31. 10.1111/jftr.12255

[B42] Moraitou D. Kolovou C. Papasozomenou C. Paschoula C. (2006). Hope and adaptation to old age: their relationship with individual-demographic factors. Soc. Indic. Res. 76, 71–93. 10.1007/s11205-005-4857-4

[B43] Morote R. Hjemdal O. Krysinska K Uribe P. M. Corveleyn J. (2017). Resilience or hope? incremental and convergent validity of the resilience scale for adults (RSA) and the Herth hope scale (HHS) in the prediction of anxiety and depression. BMC Psychol. 5, 1–13. 10.1186/s40359-017-0205-0 29078801 PMC5659010

[B44] Naci H. Ioannidis J. P. (2015). Evaluation of wellness determinants and interventions by citizen scientists. JAMA 314, 121–122. 10.1001/jama.2015.6160 26068643

[B45] Otis K. L. Huebner E. S. Hills K. J. (2016). Origins of early adolescents' hope: personality, parental attachment, and stressful life events. Can. J. Sch. Psychol. 31, 102–121. 10.1177/0829573515626715

[B46] Pomerleau K. (2020). Tax Policy and the Federal Response to COVID-19. Washington, DC: American Enterprise Institute (AEI).

[B47] Qiu J. Shen B. Zhao M. Wang Z. Xie B. Xu Y. (2020). A nationwide survey of psychological distress among Chinese people in the COVID-19 epidemic: implications and policy recommendations. Gen. Psychiatry 33:e100213. 10.1136/gpsych-2020-100213 32215365 PMC7061893

[B48] Schneider S. (2001). In search of realistic optimism. Meaning, knowledge, and warm fuzziness. Am. Psychol. 56, 250–263. 10.1037/0003-066X.56.3.250 11315251

[B49] Shaban O. S. Al-Zubi Z. Ali N. Alqotaish A. (2017). The effect of low morale and motivation on employees' productivity and competitiveness in Jordanian industrial companies. Int. Bus. Res. 10, 1–7. 10.5539/ibr.v10n7p1

[B50] Snyder C. R. Irving L. M. Anderson J. R. (1991). Hope and health, in Handbook of Social and Clinical Psychology: The Health Perspective, Vol. 15, eds Snyder C. R. Forsyth D. R. (New York, NY: Pergamon Press), 285–305.

[B51] Southwick S. M. Bonanno G. A. Masten A. S. Panter-Brick C. Yehuda R. (2014). Resilience definitions, theory, and challenges: interdisciplinary perspectives. Eur. J. Psychotraumatol. 5:25338. 10.3402/ejpt.v5.25338 25317257 PMC4185134

[B52] Thornton D. (2020). Beacons of hope: how neighborhood organizing led disaster recovery. New Engl. J. Public Policy 32:16. Available online at: https://scholarworks.umb.edu/nejpp/vol32/iss1/16/

[B53] US Army (1983). Field Manual on Leadership. Washington, DC: Department of the Army.

[B54] Wang C. J. Ng C. Y. Brook R. H. (2020). Response to COVID-19 in Taiwan: big data analytics, new technology, and proactive testing. JAMA 323, 1341–1342. 10.1001/jama.2020.3151 32125371

[B55] World Health Organization (WHO) (2019). Available online at: https://www.who.int/emergencies/diseases/novel-coronavirus-2019 (accessed August 1, 2020).

